# A quantitative evaluation of the deep learning model of segmentation and measurement of cervical spine MRI in healthy adults

**DOI:** 10.1002/acm2.14282

**Published:** 2024-01-25

**Authors:** Yifeng Zhu, Yushi Li, Kexin Wang, Jinpeng Li, Xiaodong Zhang, Yaofeng Zhang, Jialun Li, Xiaoying Wang

**Affiliations:** ^1^ Department of Radiology Peking University First Hospital Beijing China; ^2^ Department of Radiology The Second Hospital Dalian Medical University Dalian China; ^3^ School of Basic Medical Sciences Capital Medical University Beijing China; ^4^ Beijing Smart Tree Medical Technology Co. Ltd. Beijing China

**Keywords:** cervical spine, deep learning, magnetic resonance imaging, morphometrics

## Abstract

**Purpose:**

To evaluate the 3D U‐Net model for automatic segmentation and measurement of cervical spine structures using magnetic resonance (MR) images of healthy adults.

**Materials and methods:**

MR images of the cervical spine from 160 healthy adults were collected retrospectively. A previously constructed deep‐learning model was used to automatically segment anatomical structures. Segmentation and localization results were checked by experienced radiologists. Pearson's correlation analyses were conducted to examine relationships between patient and image parameters.

**Results:**

No measurement was significantly correlated with age or sex. The mean values of the areas of the subarachnoid space and spinal cord from the C2/3 (cervical spine 2–3) to C6/7 intervertebral disc levels were 102.85–358.12 mm^2^ and 53.71–110.32 mm^2^, respectively. The ratios of the areas of the spinal cord to the subarachnoid space were 0.25–0.68. The transverse and anterior‐posterior diameters of the subarachnoid space were 14.77–26.56 mm and 7.38–17.58 mm, respectively. The transverse and anterior‐posterior diameters of the spinal cord were 9.11–16.02 mm and 5.47–10.12 mm, respectively.

**Conclusion:**

A deep learning model based on 3D U‐Net automatically segmented and performed measurements on cervical spine MR images from healthy adults, paving the way for quantitative diagnosis models for spinal cord diseases.

## 1 INTRODUCTION

Cervical spondylosis (CS) is a chronic degenerative disease that is commonly present in individuals over 50 years old.^1^ CS may progress to spinal cord compression and cervical spondylotic myelopathy without apparent symptoms in the early stage. The evolution from CS to cervical spondylotic myelopathy is highly variable and difficult to predict. Some patients experience a benign form of the disease without severe symptoms, while others experience substantial deterioration over time, and their daily lives are severely affected.^2^ In clinical practice, conservative treatments are usually selected for mild and moderate CS, while surgical intervention is recommended for patients with severe intractable pain or progressive symptoms associated with neurological deficits.^3^ Therefore, precise diagnosis of CS and accurate evaluation of the severity of CS are necessary.

Magnetic resonance (MR) imaging has been widely applied in the diagnosis of cervical spine disorders because of its high soft tissue resolution.^4^ However, the current usage of cervical MR is limited to qualitative diagnosis of morphology and lacks quantitative diagnostic applications. Cervical spondylosis is one of the most common cervical spine disorders. The diagnostic criteria for cervical spondylosis include the following aspects: (1) cervical vertebral body hyperplasia, (2) cervical vertebral body slippage, (3) cervical intervertebral disc bulge, protrusion, or prolapse, (4) thickening of the posterior longitudinal ligament and/or ligamentum flavum, (5) spinal stenosis, (6) corresponding spinal cord compression and degeneration secondary to CS, and (7) other pathological conditions that should be differentiated from CS. Quantitative diagnosis can accurately assess the degree of cervical spinal stenosis, the degree of subarachnoid space and spinal cord compression, as well as the relative position of the spinal cord within the subarachnoid space, which plays an important role in the diagnosis of cervical spondylosis.

However, due to the complexity of anatomical structures (such as bones, ligaments, vessels and nerves) of the cervical spine regions and the proximity of the density and signal intensity on images, quantitative measurement of the relevant structures of cervical spine is difficult to some degree, which may prevent accurate diagnosis of CS. Effective approaches to objectively and accurately analyze the cervical structures in MR images are lacking.^5^

Artificial intelligence (AI) is an emerging technology that has been widely investigated and applied in the medical imaging field. Automatic image segmentation performed by deep learning has been achieved in the kidney, prostate and breast.^6^, ^7^, ^8^ These studies extracted the features of corresponding human tissues and organs using deep learning or radiomics methods to achieve automatic segmentation of these tissues or organs, based on which subsequent clinical tasks or goals can be achieved. Previous studies have applied AI for measurements of the cervical spine in different populations and with different modalities of imaging techniques.^9^, ^10^, ^11^, ^12^, ^13^, ^14^, ^15^

Our research group previously developed an automatic adult cervical MR segmentation model with good accuracy based on 3D U‐Net,^16^ but studies of its application in practice (e.g., measurement of various structures of the cervical spine, etc.) have not been conducted. In this study, we used this constructed deep learning model to automatically segment and measure the various structures of the cervical spine, and we also propose some new definitions for measuring the subarachnoid space and spinal cord.

## 2 METHODS

### 2.1 Patients

This research was approved by the Ethics Review Committee of Peking University First Hospital. ([2019(170)]). The need to obtain written informed consent from subjects was waived by the Ethics Review Committee. MR images of suspected CS patients from March 2016 to April 2022 were collected retrospectively from the picture archiving and communication system in our hospital. Two experienced radiologists with more than 15 years of experience checked all enrolled cases to confirm the absence of abnormalities and excluded cases using the following exclusion criteria: (1) herniation of intervertebral discs in the cervical MRI; (2) presence of organic lesions in the cervical spine; and (3) poor image quality.

Finally, a total of 160 subjects were enrolled in this study (60 males and 100 females). The ages of patients ranged from 18 to 82 (37.7 ± 13.5) years. A total of 480 MRI series (sagittal T_1_WI, sagittal T_2_WI and axial T_2_WI; 160 of each) were collected. The 3D U‐Net model predicted 1189 vertebral bodies (including 960 cervical vertebral bodies and 66 thoracic vertebral bodies) and 866 intervertebral discs (including 800 cervical intervertebral discs and 66 thoracic intervertebral discs).

### 2.2 MRI equipment and technology

The MRI scanning equipment included Signa Excite 3.0T (GE Medical Systems, GE Medical Systems, Chicago, IL), Discovery HD 750 3.0T (GE Medical Systems), Aera 1.5T (Siemens Healthcare Engineers, Siemens Healthcare Engineers, Erlangen, Germany), Ingenia 3.0T (Philips Medical Systems, Philips Medical Systems, Achieva, The Netherlands), Achieva 3.0T (Philips Medical Systems), Multiva 1.5T (Philips Medical Systems) and uMR 790 3.0T (United Imaging Healthcare, United Imaging Healthcare, Shanghai, China). The scanning sequences included conventional sagittal T1WI, T2WI, axial T2WI and fat suppression T_2_WI (FS‐T_2_WI). Supplementary Table 1 lists the sequence and parameters of MRI.

### 2.3 Image segmentation

The DICOM images of sagittal T_1_WI, T_2_WI and axial T_2_WI of the cervical spine were converted into NiFTI format. A previously constructed 3D U‐Net model for cervical MR segmentation and measurement was used to predict the structures of the subarachnoid space and spinal cord in axial T_2_WI and the structures of the cervical spine column and intervertebral discs in sagittal T_1_WI and T_2_WI.^16^ The labels predicted by the model were judged by a radiologist specializing in diagnostic medical imaging of bone and joints with experience ≥15 years, whose judgment was used as the ground truth. If the predicted labels have a high degree of overlap with the corresponding anatomical structures, the radiologist will consider the model prediction results to be satisfactory. The cervical spine column and intervertebral discs were evaluated in midsagittal T_2_WI, and the subarachnoid space and spinal cord were evaluated in axial T_2_WI. The results of segmentation were output after the model segmentation was completed, which met the requirements of radiologists for diagnosis, for example, the segmentation results were consistent with the actual anatomical structure locations. The inaccurate model segmentation results were modified to ensure meeting the needs before measurements. About 90% of the model segmentation results did not need to be modified. The amount of work required to modify the model segmentation results was minimal and simple, and each modification took less than a minute.

**FIGURE 1:**
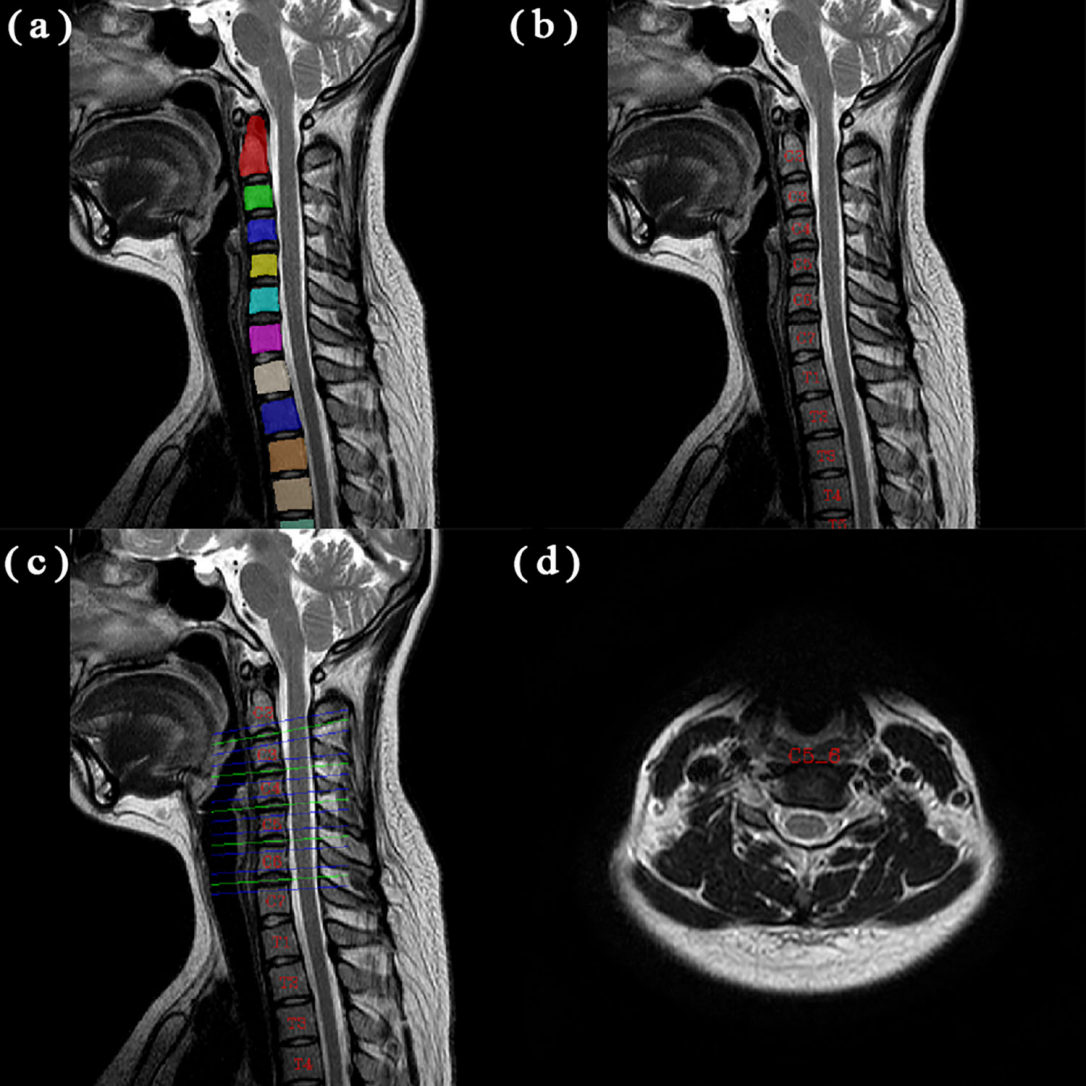
Localization process of the vertebral body and intervertebral discs. (a) The vertebral body is segmented on the sagittal T_2_WI image. (b) The position of each vertebral body from C2 to C7 is determined in sequence. (c) The measurement slices of intervertebral discs are located. The blue and green lines represent the axial T_2_WI scan plane. The green lines passing through the intervertebral disc level indicate the measurement plane. (d) The positioning information of the sagittal T_2_WI is matched to the axial T_2_WI to obtain the localization of each intervertebral disc (the C5/6 intervertebral disc is shown here for illustration).

After segmentation, the vertebral body was automatically located according to the sagittal vertebral body segmentation results. The location information of sagittal T_2_WI was matched with that in axial T_2_WI, and the location of each intervertebral disc was obtained (Figure 1).

### 2.4 Definition of measurements of the subarachnoid space and spinal cord

**FIGURE 2:**
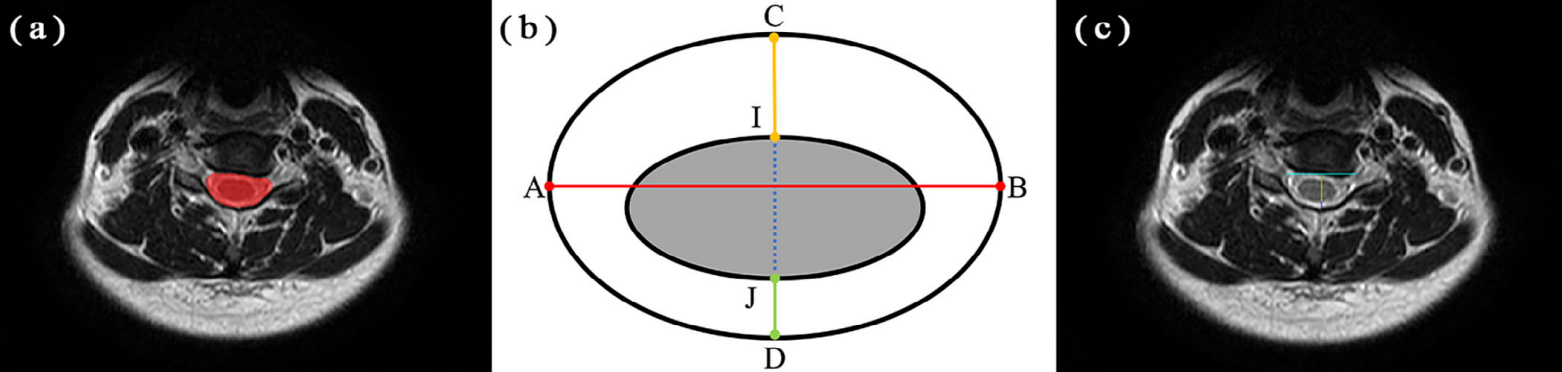
Schematic diagram of subarachnoid space measurement. (a) Segmentation of the subarachnoid space in axial T_2_WI is shown in the red area. (b) Measurement of diameter lines of the subarachnoid space are depicted. AB represents the transverse diameter of the subarachnoid space, CD represents the anterior‐posterior diameter, CI represents the anterior extraspinal space, and JD represents the posterior extraspinal space. (c) A visualization of diameter lines of the subarachnoid space.

**FIGURE 3:**
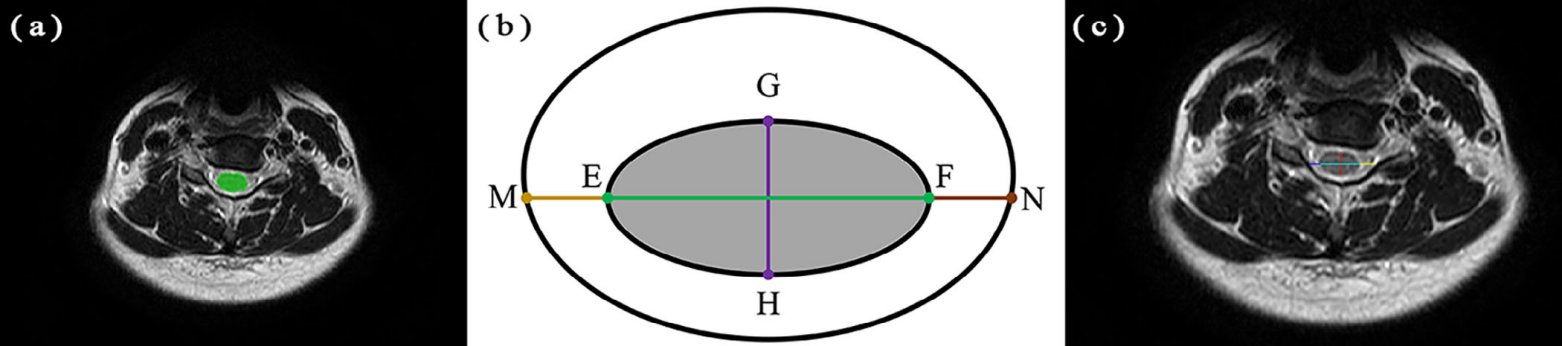
Schematic diagram of measurements of the spinal cord. (a) Segmentation of the spinal cord in axial T_2_WI is shown in green. (b) Measurement of the diameter lines of the spinal cord are depicted. EF represents the transverse diameter of the spinal cord. GH represents the anterior‐posterior diameter. ME represents the right extraspinal space, and FN represents the left extraspinal space. (c) A visualization of the diameter lines of the spinal cord.

After localization of the intervertebral disc, axial T_2_WIs from the C2/3 (cervical spine 2−3) to C6/7 intervertebral discs were used for automatic measurements. The measurements included area, transverse diameter, anterior‐posterior diameter of the subarachnoid space and spinal cord and lengths of the anterior, posterior, left and right spaces of the spinal cord (Figures 2 and 3). The specific definitions of these measurements are presented in Table 1.

**TABLE 1:** Definition of measurements of the subarachnoid space and spinal cord.

Definition	Interpretation
Transverse diameter of subarachnoid space (AB)	Distance between the right and left boundaries of the labeled of subarachnoid space
Anterior‐posterior diameter of subarachnoid space (CD)	Distance between the anterior and posterior boundaries of subarachnoid space at the midpoint of AB
Transverse diameter of spinal cord (EF)	Distance between the right and left boundaries of spinal cord
Anterior‐posterior diameter of spinal cord (GH)	Distance between the anterior and posterior boundaries of spinal cord at the midpoint of EF
Anterior extraspinal space (CI)	I point is the intersection of CD and anterior spinal cord
Posterior extraspinal space (JD)	J point is the intersection point between CD and posterior edge of spinal cord
Right extraspinal space (ME)	M point is the intersection of EF and the outermost edge on the right side of subarachnoid space
Left extraspinal space (FN)	N point is the intersection point of EF and the left outermost edge of subarachnoid space

### 2.5 Statistical analysis

Statistical analyses were performed by the Statistical Product and Service Software Automatically (SPSSAU) program (https://spssau.com/)^17^ and R 4.2.0. Numeric variables were expressed as the mean ± SD. Pearson's correlation analyses were conducted to examine the relationship between sex, age and measurement results and the correlations between the spinal cord area and extraspinal space as well as between the subarachnoid space and extraspinal space. Welch's ANOVA was conducted to evaluate the differences in the same measurements across different intervertebral disc levels. Paired t‐tests were used to compare the variations in measurements between different intervertebral levels. *P* values less than 0.05 were considered statistically significant.

## 3 RESULTS

### 3.1 Correlation test between sex, age and measurement results

All measurement results were normally distributed. Pearson's correlation analysis did not show any significant correlation between the measurement results and age or sex (Supplementary material).

### 3.2 Measurement results of the subarachnoid space and spinal cord

**FIGURE 4:**
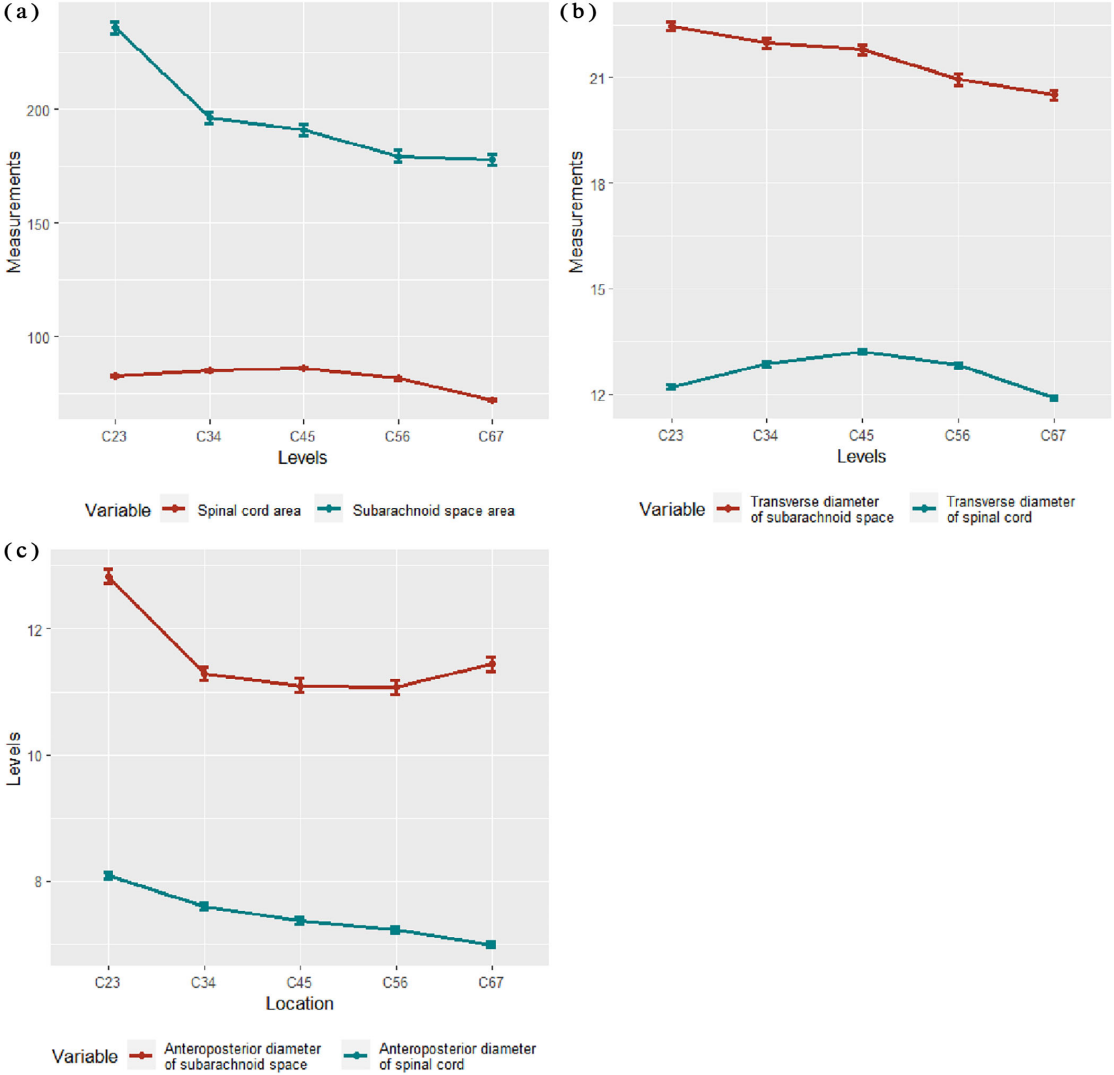
The changing trends in the measurements of subarachnoid space and spinal cord at different levels of intervertebral discs. (a) Area of the subarachnoid space and spinal cord. The area of the spinal cord increased slightly from C2/3 to C4/5 level and gradually decreased from C4/5 to C6/7 level. The area of subarachnoid space gradually decreased from C2/3 to C6/7 level, with a decrease from C2/3 to C3/4 level; (b) transverse diameter of the subarachnoid space and spinal cord. The transverse diameter of the spinal cord gradually increased from C2/3 to C4/5 level and gradually decreased from C4/5 to C6/7 level; this trend was roughly symmetrically distributed centered on C4/5 level. The transverse diameter of the subarachnoid space gradually decreased from C2/3 to C6/7 level; (c) anterior‐posterior diameter of the subarachnoid space and spinal cord. The anterior‐posterior diameter of the spinal cord gradually decreased from C2/3 to C6/7 level. > The anterior‐posterior diameter of the subarachnoid space gradually decreased from C2/3 to C4/5 level, with a particularly significant decrease from C2/3 to C3/4 level and a gradual increase from C4/5 to C6/7 level.

The measurement results of the subarachnoid space and spinal cord at each level of intervertebral discs are shown in Table 2. The changing trends in measurement indicators at different levels of the intervertebral discs are shown in Figure 4. In the cervical spine MR of 160 healthy adults, the area of the subarachnoid space from the C2/3–C6/7 level ranged from 102.85 mm^2^ to 358.12 mm^2^, and the area of the spinal cord ranged from 53.71 mm^2^ to 110.32 mm^2^. The transverse diameter of the subarachnoid space ranged from 14.77 mm to 26.56 mm, and the transverse diameter of the spinal cord ranged from 7.38 mm to 17.58 mm. The anterior‐posterior diameter of the subarachnoid space ranged from 7.38 mm to 17.58 mm, and the anterior‐posterior diameter of the spinal cord ranged from 5.47 mm to 10.12 mm.

**TABLE 2:** Measurement results of the subarachnoid space and spinal cord at each level of the intervertebral disc.

Location of intervertebral disc	Subarachnoid space area (mm^2^)	Spinal cord area (mm^2^)	Transverse diameter of subarachnoid space (mm)	Transverse diameter of spinal cord (mm)	Anterior‐posterior diameter of subarachnoid space (mm)	Anterior‐posterior diameter of spinal cord (mm)
C2/3	235.91 ± 34.17	82.52 ± 7.59	22.45 ± 1.69	12.21 ± 0.87	12.84 ± 1.47	8.09 ± 0.69
C3/4	196.32 ± 30.26	85.20 ± 7.52	21.98 ± 1.75	12.85 ± 0.87	11.29 ± 1.30	7.59 ± 0.66
C4/5	190.95 ± 30.55	86.10 ± 7.59	21.79 ± 1.79	13.20 ± 0.82	11.10 ± 1.44	7.36 ± 0.69
C5/6	179.51 ± 32.79	81.50 ± 8.23	20.95 ± 2.14	12.82 ± 0.89	11.07 ± 1.40	7.22 ± 0.67
C6/7	178.03 ± 31.48	72.02 ± 8.05	20.50 ± 1.86	11.89 ± 0.88	11.44 ± 1.40	6.99 ± 0.57

*Note*: Data are presented as the mean ± SD.

Figure 4 shows the changing trends in the measurements of the subarachnoid space and spinal cord at different levels of intervertebral discs. The area of the spinal cord increased slightly from the C2/3 to C4/5 level and gradually decreased from the C4/5 to C6/7 level. The area of the subarachnoid space gradually decreased from the C2/3 to C6/7 level, with a decrease from the C2/3 to C3/4 level. The transverse diameter of the spinal cord gradually increased from the C2/3 to C4/5 level and gradually decreased from the C4/5 to C6/7 level; this trend was roughly symmetrically distributed centered on the C4/5 level. The transverse diameter of the subarachnoid space gradually decreased from the C2/3 to C6/7 level. The anterior‐posterior diameter of the spinal cord gradually decreased from the C2/3 to C6/7 level. The anterior‐posterior diameter of the subarachnoid space gradually decreases from the C2/3 to C4/5 level, with a particularly significant decrease from the C2/3 to C3/4 level and a gradual increase from the C4/5 to C6/7 level. The rates of measurement indicators at different levels of intervertebral discs are presented in Table 3.

**TABLE 3:** Measurement indicators at different levels of intervertebral discs.

	C2/3	C3/4	C4/5	C5/6	C6/7
ASS/PSS	1.21 (0.88)	0.91 (0.56)	0.84 (0.48)	1.08 (0.74)	1.75 (1.59)
SCA/SSA	0.36 (0.05)	0.44 (0.06)	0.46 (0.06)	0.47 (0.07)	0.41 (0.07)
TDSC/TDSS	0.55 (0.04)	0.59 (0.05)	0.61 (0.05)	0.62 (0.06)	0.58 (0.06)
ADSC/ADSS	0.63 (0.06)	0.68 (0.06)	0.67 (0.07)	0.66 (0.07)	0.62 (0.07)
RSS/LSS	1.01 (0.34)	1.05 (0.54)	0.99 (0.40)	0.99 (0.41)	0.96 (0.36)

*Note*: Data are presented as the mean (SD). ASS/PSS is the ratio of anterior to posterior extra spinal space. TDSC/TDSS is the ratio of transverse diameter of subarachnoid space to transverse diameter of spinal cord. ADSC/ADSS is the ratio of anterior‐posterior diameter of spinal cord to anterior‐posterior diameter of subarachnoid space. SCA/SSA is the ratio of spinal cord area to subarachnoid space area. RSS/LSS is the ratio of right extraspinal space to left extraspinal space.

**FIGURE 5:**
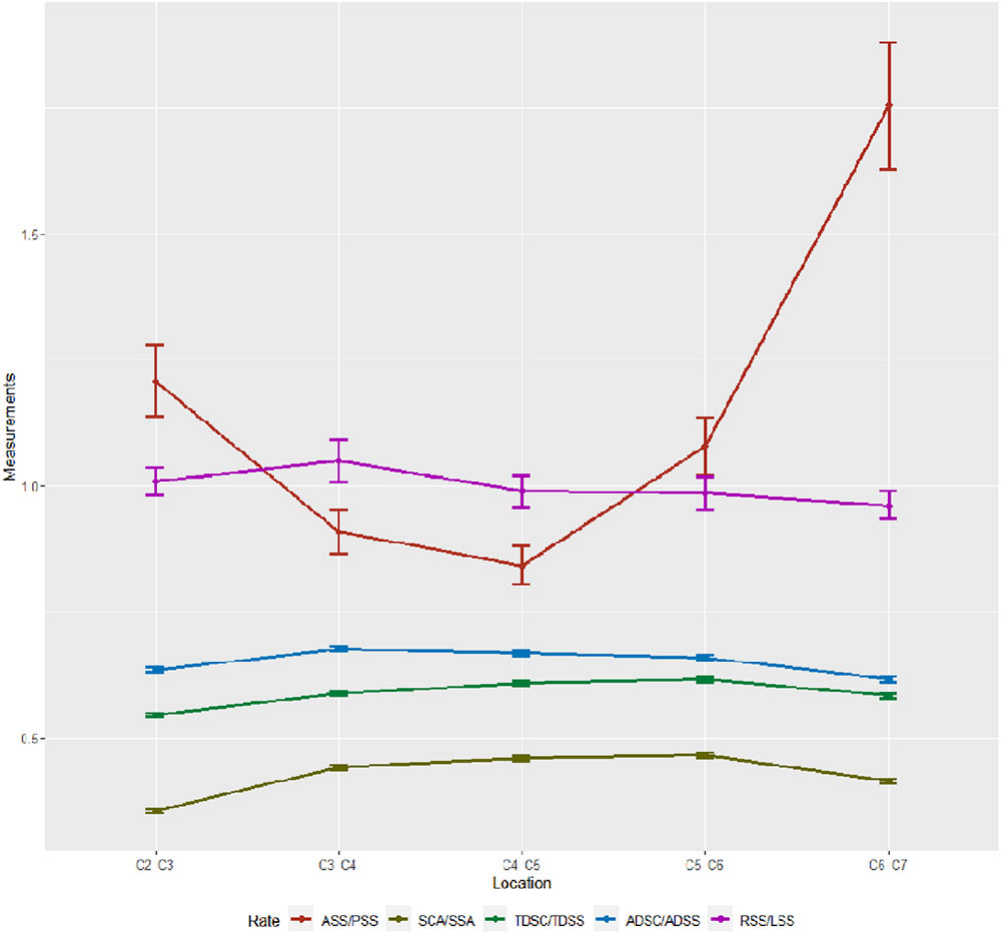
The changing trends in the rates of measurement indicators at different levels of intervertebral discs. The ratio of anterior to posterior extraspinal space (ASS/PSS) gradually decreased from the C2/3 to C4/5 level and significantly increased from the C4/5 to C6/7 level. The ratio of the transverse diameter of the subarachnoid space to the transverse diameter of the spinal cord (TDSC/TDSS), the ratio of the anterior‐posterior diameter of the spinal cord to the anterior‐posterior diameter of the subarachnoid space (ADSC/ADSS) and the ratio of the spinal cord area to the subarachnoid space area (SCA/SSA) showed similar patterns of changes. They all gradually increased from the C2/3 to C4/5 level and decreased from the C4/5 to C6/7 level, without any significant overall change. The ratio of the right extraspinal space to the left extraspinal space (RSS/LSS) gradually increased from the C2/3 to C3/4 level and gradually decreased from the C3/4 to C6/7 level, without any significant overall change. ASS: anterior extraspinal space, PSS: posterior extraspinal space, SCA: spinal cord area, SSA: subarachnoid space area, TDSC: transverse diameter of subarachnoid space, TDSS: transverse diameter of spinal cord, ADSC: anterior‐posterior diameter of spinal cord, ADSS: anterior‐posterior diameter of subarachnoid space, RSS: right extraspinal space, LSS: left extraspinal space.

The changing trends in the rates of measurement indicators at different levels of intervertebral discs are presented in Figure 5. The ratio of anterior to posterior extraspinal space (ASS/PSS) gradually decreased from the C2/3 to C4/5 level and significantly increased from the C4/5 to C6/7 level. The ratio of the transverse diameter of the subarachnoid space to the transverse diameter of the spinal cord (TDSC/TDSS), the ratio of the anterior‐posterior diameter of the spinal cord to the anterior‐posterior diameter of the subarachnoid space (ADSC/ADSS) and the ratio of the spinal cord area to the subarachnoid space area (SCA/SSA) showed similar patterns of changes. They all gradually increased from the C2/3 to C4/5 level and decreased from the C4/5 to C6/7 level, without any significant overall change. The ratio of the right extraspinal space to the left extraspinal space (RSS/LSS) gradually increased from the C2/3 to C3/4 level and gradually decreased from the C3/4 to C6/7 level, without any significant overall change.

### 3.3 Measurement results of the extraspinal space

**FIGURE 6:**
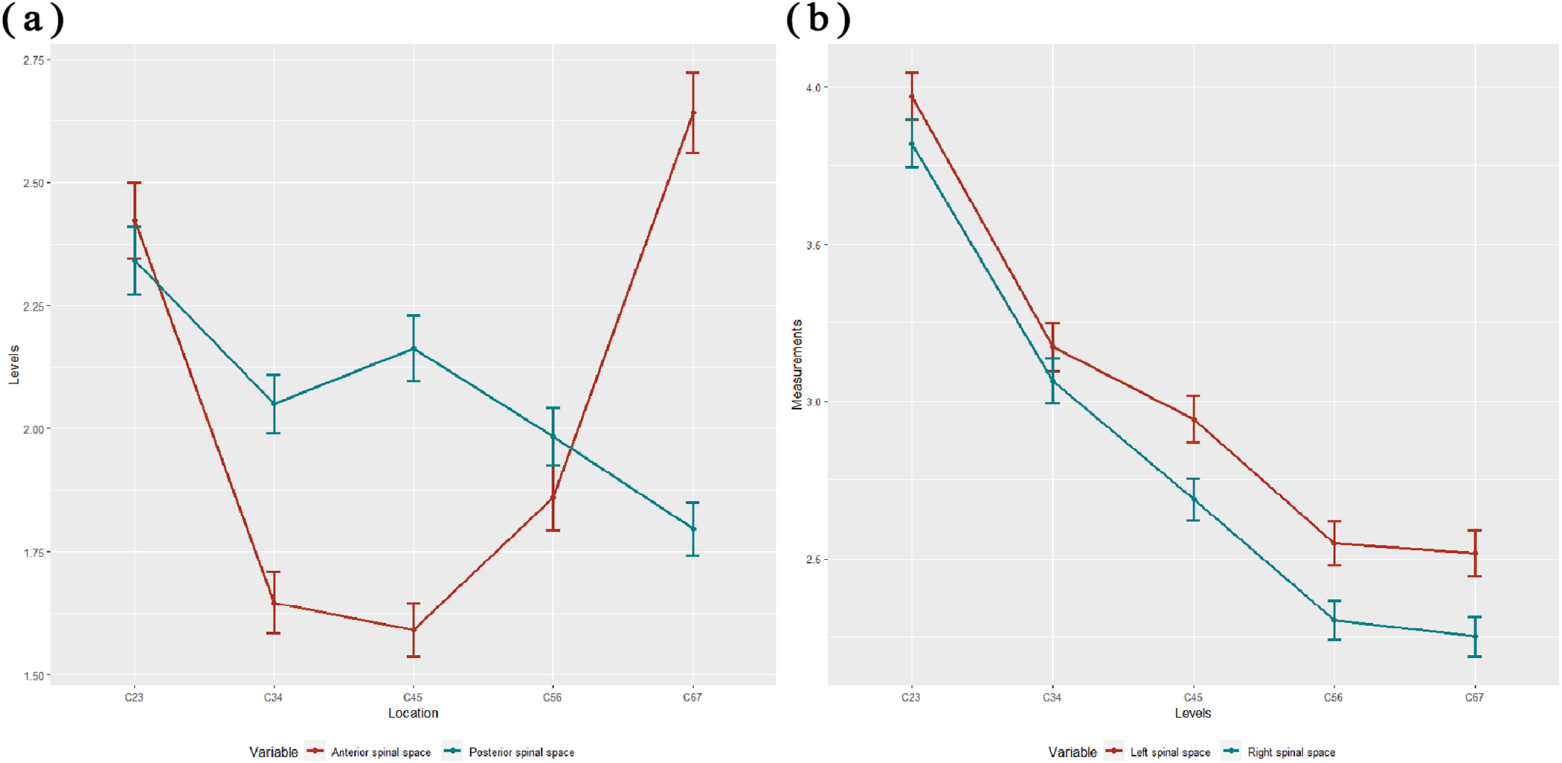
The changing trends in the measurements of extraspinal space at different levels of intervertebral discs. (a) Anterior extraspinal space (red line) and posterior extraspinal space (green line). The anterior spinal cord space (ASS) decreased from C2/3 to C4/5 and increased from C4/5 to C6/7, with a decrease from C2/3 to C3/4 and a relatively small decrease from C3/4 to C4/5. The posterior spinal cord space (PSS) decreased from C2/3 to C3/4, slightly increased from C3/4 to C4/5 but did not exceed the level of C2/3, and gradually decreased from C4/5 to C6/7; (b) left extraspinal space (red line) and right extraspinal space (green line). The left extraspinal space (LSS) and the right extraspinal space (RSS) had similar patterns of change at different disc levels: a gradual decrease from C2/3 to C6/7, a decrease from C2/3 to C3/4 and a relatively small decrease from C5/6 to C6/7.

The measurement results of the extraspinal space at each level of the intervertebral discs are presented in Table 4 and Figure 6. The anterior extraspinal space (ASS) ranged from 0.35 mm to 5.63 mm, and the posterior extraspinal space ranged from 0.30 mm to 5.98 mm. The left extraspinal space ranged from 0.35 mm to 6.68 mm, and the right extraspinal space ranged from 0.70 mm to 6.25 mm.

**TABLE 4:** Measurement results of the extraspinal space at each level of the intervertebral disc.

Location	Anterior extraspinal space (mm)	Posterior extraspinal space (mm)	Left extraspinal space (mm)	Right extraspinal space (mm)
C2/3	2.42 ± 0.98	2.34 ± 0.87	3.97 ± 0.95	3.82 ± 0.96
C3/4	1.65 ± 0.79	2.05 ± 0.75	3.17 ± 0.97	3.07 ± 0.90
C4/5	1.59 ± 0.69	2.16 ± 0.84	2.94 ± 0.94	2.69 ± 0.83
C5/6	1.86 ± 0.84	1.98 ± 0.73	2.55 ± 0.89	2.30 ± 0.80
C6/7	2.64 ± 1.02	1.80 ± 0.68	2.52 ± 0.92	2.25 ± 0.80

*Note*: Data presented are the mean ± SD.

The ASS decreased from the C2/3 to C4/5 level and increased from the C4/5 to C6/7 level, with a significant decrease from the C2/3 to C3/4 level and a relatively small decrease from the C3/4 to C4/5 level. The posterior extraspinal space (PSS) decreased from the C2/3 to C3/4 level, increased slightly from the C3/4 to C4/5 level (not exceeding the size of C2/3) and gradually decreased from the C4/5 to C6/7 level. The changes in the left extraspinal space (LSS) and right extraspinal space (RSS) at different disk levels showed the following similar patterns. They gradually decreased from the C2/3 to C6/7 level, with a significant decrease from the C2/3 to C3/4 level and a relatively small decrease from the C5/6 to C6/7 level.

### 3.4 Welch's ANOVA for the same measurement at different intervertebral disc levels

Welch's ANOVA showed that all measurements had statistically significant differences at different intervertebral disc levels (Table 5). The corresponding ridge map is presented in Supplementary Figure 1.

**TABLE 5:** Results of ANOVA between location and measurements (mean ± SD).

	Location						
Items	C2−3	C3−4	C4−5	C5−6	C6−7	Welch F	*P*
Subarachnoid space area	235.91 ± 34.17	196.32 ± 30.26	190.95 ± 30.55	179.51 ± 32.79	178.03 ± 31.48	78.266	0.000^**^
Spinal cord area	82.52 ± 7.59	85.20 ± 7.52	86.10 ± 7.59	81.50 ± 8.23	72.02 ± 8.05	80.048	0.000^**^
Ratio of spinal cord area to subarachnoid space area	0.36 ± 0.05	0.44 ± 0.06	0.46 ± 0.06	0.47 ± 0.07	0.41 ± 0.07	104.956	0.000^**^
Transverse diameter of subarachnoid space	22.45 ± 1.69	21.98 ± 1.75	21.79 ± 1.79	20.95 ± 2.14	20.50 ± 1.86	29.916	0.000^**^
Anterior‐posterior diameter of subarachnoid space	12.84 ± 1.47	11.29 ± 1.30	11.10 ± 1.44	11.07 ± 1.40	11.44 ± 1.40	40.888	0.000^**^
Transverse diameter of spinal cord	12.21 ± 0.87	12.85 ± 0.87	13.20 ± 0.82	12.82 ± 0.89	11.89 ± 0.88	61.223	0.000^**^
Anterior‐posterior diameter of spinal cord	8.09 ± 0.69	7.59 ± 0.66	7.36 ± 0.69	7.22 ± 0.67	6.99 ± 0.57	67.154	0.000^**^
Anterior extraspinal space	2.42 ± 0.98	1.65 ± 0.79	1.59 ± 0.69	1.86 ± 0.84	2.64 ± 1.02	44.466	0.000^**^
Posterior extraspinal space	2.34 ± 0.87	2.05 ± 0.75	2.16 ± 0.84	1.98 ± 0.73	1.80 ± 0.68	10.971	0.000^**^
Right spinal cord space	3.82 ± 0.96	3.07 ± 0.90	2.69 ± 0.83	2.30 ± 0.80	2.25 ± 0.80	82.451	0.000^**^
Left space of spinal cord	3.97 ± 0.95	3.17 ± 0.97	2.94 ± 0.94	2.55 ± 0.89	2.52 ± 0.92	64.615	0.000^**^

*Note*: Data presented are mean ± SD.

** *P* < 0.01.

### 3.5 Symmetry test in the extraspinal space

The results of the t‐tests at all levels are presented in Table 6. The differences between the anterior and posterior extraspinal space in C3/4, C4/5 and C6/7 were statistically significant (*P* < 0.05). The differences between the right and left extraspinal space in C4/5, C5/6 and C6/7 were statistically significant (*P* < 0.05). Both the posterior spinal cord space and the left and right spinal cord spaces were asymmetrical at C2−3, while the anterior spinal cord space was asymmetrical at C6−7.

**TABLE 6:** *t*‐Tests of extraspinal space.

	Anterior‐posterior symmetry			Right‐left symmetry		
	Anterior extraspinal space (mm)	Posterior extraspinal space (mm)	*P*	Right extraspinal space (mm)	Left extraspinal space (mm)	*P*
C2/3	2.42 (0.98)	2.34 (0.88)	0.953	3.97 (0.95)	3.82 (0.96)	0.163
C3/4	1.65 (0.79)	2.05 (0.75)	0.008	3.17 (0.97)	3.07 (0.90)	0.306
C4/5	1.59 (0.69)	2.16 (0.84)	<0.001	2.94 (0.94)	2.69 (0.83)	0.010
C5/6	1.86 (0.84)	1.98 (0.73)	0.224	2.55 (0.89)	2.30 (0.80)	0.010
C6/7	2.64 (1.02)	1.80 (0.68)	<0.001	2.52 (0.92)	2.25 (0.80)	0.006

*Note*: Data are presented as the mean (SD).

Scatter plots and the smooth curves fitted by the generalized additive model of the anterior‐posterior interspace and the left‐right interspace are presented in Supplementary Figure 2. None of the plots showed clear symmetry or conformed to a simple linear correlation.

## 4 DISCUSSION

In the current study, we evaluated and used a segmentation model developed before and performed a measurement algorithm of cervical spine MR structures in healthy adults using a deep learning model based on 3D U‐Net. Because of the high soft tissue resolution, MRI can clearly display soft tissue structures and provide quantitative imaging information for the diagnosis of CS. Objective measurements for cervical spine MRI are important for the diagnosis of cervical spondylosis, and radiologists seldom measure quantitative indicators for cervical spine MRI in daily work because manual measurement of cervical spine structures is time‐consuming. 3D U‐Net is a well‐established model that has been widely applied in clinical studies. In this study, we evaluated the feasibility of its application in cervical spine MR. Results revealed that, in clinical practice, automated quantitative measurements of key structures in the cervical spine can be accomplished using the deep learning approach, which is clearly timesaving compared with manual measurements by radiologists. According to our own actual work and experience, measuring these values in clinical practice usually takes 5−10 min in daily work, whereas the measurement time of the model is negligible using the deep learning approach. The deep learning model developed by preceding studies can automatically generate objective MRI reports, which has potential clinical significance in the diagnosis of CS.

Our results did not reveal any significant correlation between any measurement result and age or sex. This result suggests that the structures of the cervical spine appear to be stable in adulthood and are unlikely to be influenced by age. The absence of a correlation with sex may be because the nervous system is not a target organ for sex hormones.

Our results also demonstrated that the area of the subarachnoid space, anterior‐posterior diameter and transverse diameter of the subarachnoid space gradually decreased as the level of the intervertebral disc increased. This trend is possibly related to the continuation from the medulla oblongata. Due to the presence of cervical enlargement, the area of the spinal cord reaches its maximum at the level of the C4−5 intervertebral disc, and the ratio of the area of the spinal cord to the area of the subarachnoid space and transverse diameter of the spinal cord also reaches its maximum at this level. The anterior‐posterior diameter of the spinal cord showed a decreasing trend with the increase in the intervertebral disc level, which may be because cervical enlargement mainly manifests in the left and right diameter direction of the spinal cord. This also explains why the anterior space of the spinal cord has a minimum value at the C4−5 level, while other extraspinal spaces showed a decreasing trend with the increase in intervertebral disc level. We also observed that a larger area of subarachnoid space is often accompanied by a larger area of extraspinal space. A larger area of the spinal cord is often accompanied by a smaller ASS and a larger area in the remaining extraspinal space.

The segmentation model is just a tool used in this study. We used this segmentation model to make detailed measurements of the structure of the cervical spine, which can serve as a baseline and statistical trend analysis for the normal population. Although some of the existing studies have investigated the segmentation models of cervical spine MR structures, there has been no exploration of baseline reference standards for the normal population. Some previous modeling studies have proposed several objective measurements of cervical spine structures in cervical spine X‐ray,^9^, ^10^ CT,^9^, ^11^ and MRI.^12^, ^13^, ^14^, ^15^ Most previous studies focused on X‐ray and CT. As X‐ray and CT emphasize on measurements of bony structures in the cervical spine, such as bony spinal canal and vertebral tuberosity, measurements of structures within the spinal canal have not been well addressed. Peabody et al. mentioned that the average diameter of the cervical vertebrae is 17 mm.^18^ This finding differs from the measurements in our study because the diameter of bony spinal canal is larger than the diameter of subarachnoid space. The boundaries of bony spinal canal are usually well defined on cervical X‐ray or CT, but often poorly defined on cervical spine MRI. X‐ray and CT images of the cervical spine were not included in this study. Therefore, the boundary of subarachnoid space was used as the boundary of cervical spinal canal, and our measurements were smaller than those mentioned above.

Some previous studies also developed objective measurements of cervical spine structures in cervical spine MRI. Sherman et al.^12^ measured the anterior‐posterior and transverse diameters of the spinal cord at each vertebral level and computed the simple product of these diameters to provide a single useful numerical value termed the approximate cord area. The results revealed a cervical spine enlargement ranging from C4 to C6, reaching a peak at the C4 level. Our automatic measurement results are generally consistent with those reported in Sherman et al.’s study. Ulbrich et al.^13^ examined cervical spine MRI in 140 healthy volunteers. The midsagittal diameters and areas of the spinal canal and spinal cord were measured at the midvertebral levels of C1, C3, and C6. Differences were revealed in sex, spinal level, interaction between sex and spinal level, and body height. Ulbrich et al. reported that age had a significant yet limited influence on these measurements, which is similar to our results as well as another study.^14^ In the study by Ruegg et al.,^15^ the authors measured the transverse spinal canal and cord area, the transverse and sagittal cord diameter, and the sagittal canal diameter of the cervical spine on axial T_2_WI MR images between patients with acute cervical spinal cord injury (CSCI) and healthy adults. Our study has similar results regarding the subarachnoid space area, spinal cord area and the ratio of spinal cord area to subarachnoid space area in healthy adults compared with those in Ruegg et al.’s study.

In addition, our work proposed some new definitions for measuring the subarachnoid space and spinal cord using the deep learning models, especially for four extraspinal spaces based on the previous segmentation model of cervical spine, which have not been previously described and can be used for quantitative diagnosis of spinal cord‐related diseases. These indicators that we proposed may potentially be useful in the diagnosis of diseases. However, since there was no deep learning method in the past, it is not easy to measure so many indicators using manual measurement methods. Therefore, we proposed a method for measuring these indicators based on the deep learning method. Measuring the extraspinal space can be used to judge lateral deviation or local compression of the spinal cord for diagnosing local spinal stenosis. The diameter and area of the spinal cord are related to spinal cord diseases, such as spinal cord atrophy or tumors. Furthermore, if cervical disc herniation leads to compression of the subarachnoid space, the anterior space of spinal cord will be narrowed. If there is an extramedullary subdural tumor or spinal cord malformation within the spinal canal, the anterior space of spinal cord may be widened posteriorly.

The anterior extraspinal interspace is more sensitive to intervertebral disc herniation and ligamentous thickening than several other interspaces. The anterior spinal interspace from C2‐3 to C4‐5 suddenly decreases. If there is a lesion in the anterior aspect of the spinal canal (e.g., mild herniation of a cervical disc), the spinal cord would easily be compressed because a neck bulge occurs in this location. However, as the intervertebral disc level decreases, the anterior spinal cord space becomes wider, while the posterior spinal cord space becomes narrower. Therefore, if there is a lesion in the posterior part of the spinal canal, the spinal cord is also susceptible to compression. If the cervical posterior longitudinal ligament is thickened at the superior level, it has less effect on the spinal cord, while it has a greater effect at the inferior level. The anterior and posterior extramedullary spaces are significantly abnormal in diseases such as Hiruzen's disease with high sensitivity, which has clinical significance. Most of these metrics above were developed based on the manual measurements of metrics from the past. We can now measure these metrics automatically using deep learning methods, and we believe that these metrics are accurate. However, comparison with the diagnostic efficacy of radiologists, which is one of our next steps, was not the focus of the current study.

From a technical point of view, this study still needs more data for model iteration in the future to improve the generalization ability and robustness of the model. As this study included data only from healthy adults, we need to incorporate more abnormal MRI data of cervical spine and spinal cord disorders to establish an automated quantitative diagnostic model for cervical spine disease. In addition, further studies investigating disease representations and diagnostic accuracy using deep learning quantitative evaluation are currently in progress.

## 5 CONCLUSION

In summary, we used a 3D U‐Net deep learning model to automatically measure the diameter of the subarachnoid space and spinal cord in the MRI of healthy adults. We also proposed indicators for the measurement of subarachnoid space and spinal cord diameter, paving the way for the establishment of a quantitative diagnosis model of cervical and spinal cord diseases in the future.

## AUTHOR CONTRIBUTION

Guarantor of integrity of the entire study: Xiaoying Wang. Study concepts and design: Yifeng Zhu, Xiaoying Wang, Xiaodong Zhang. Literature research: Yifeng Zhu, Yushi Li. Clinical studies: Jinpeng Li, Yaofeng Zhang, Jialun Li. Experimental studies/data analysis: Kexin Wang, Yaofeng Zhang, Jialun Li. Statistical analysis: Kexin Wang, Jinpeng Li, Yifeng Zhu. Manuscript preparation: Yifeng Zhu, Yushi Li. Manuscript editing: Yifeng Zhu, Yushi Li, Kexin Wang, Xiaoying Wang.

## CONFLICT OF INTEREST STATEMENT

The authors declare no conflicts of interest.

## ETHICS APPROVAL AND CONSENT TO PARTICIPATE

The study was approved by the ethics committee of Peking University First Hospital ([2019(170)]), which waived the need to obtain written informed consent from the included patients. All procedures performed in studies involving human participants were in accordance with the ethical standards of the institutional and/or national research committee and with the 1964 Helsinki declaration and its later amendments or comparable ethical standards.

## CONSENT FOR PUBLICATION

Not applicable.

## Supporting information

Supporting Information

Supporting Information

## Data Availability

The datasets used during the current study are available from the corresponding author on reasonable request.
