# Supplementary material for: A quantitative evaluation of the deep learning model of segmentation and measurement of cervical spine MRI in healthy adults
Source: J Appl Clin Med Phys. 2024 Jan 25;25(3):e14282. doi: 10.1002/acm2.14282 (PMC10930005; doi:10.1002/acm2.14282)
Supplement: Supplementary file 1 — Supporting Information [file ACM2-25-e14282-s002.docx]

**Supplementary Fig. 1** Ridge map of measurement results at different levels of the spinal cord. All measurements showed statistically significant differences at different intervertebral disc levels. (A) Anterior extraspinal space; (B) posterior extraspinal space; (C) left extraspinal space; (D) right extraspinal space; (E) spinal cord area; (F) subarachnoid space area; (G) anterior-posterior diameter of the spinal cord; (H) transverse diameter of the spinal cord; (I) transverse diameter of the subarachnoid space; (J) anterior-posterior diameter of the subarachnoid space.

**﻿** **
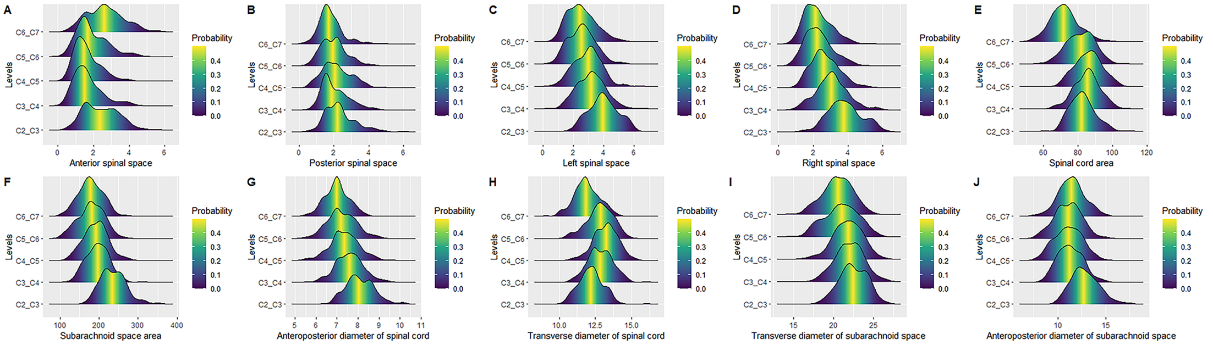
**

**Supplementary Fig. 2** Scatter plots and smooth curves fitted by the generalized additive model of the anterior-posterior spinal space and the left-right spinal space. The colors of the points and lines in the graph represent different levels of intervertebral discs. (A) Scatter plots of the anterior-posterior interspace. The anterior spinal cord space was asymmetrical at C6-7, while the posterior spinal cord space was asymmetrical at C2-3. (B) Smooth curves fitted by the generalized additive model of the anterior-posterior spinal cord space. None of the above plots show clear symmetry or conform to a simple linear correlation. (C) Scatter plots of the left-right spinal space. (D) Smooth curves fitted by the generalized additive model of the left-right spinal cord space. None of the above plots show clear symmetry or conform to a simple linear correlation.
