# Supplementary material for: A quantitative evaluation of the deep learning model of segmentation and measurement of cervical spine MRI in healthy adults
Source: J Appl Clin Med Phys. 2024 Jan 25;25(3):e14282. doi: 10.1002/acm2.14282 (PMC10930005; doi:10.1002/acm2.14282)
Supplement: Supplementary file 2 — Supporting Information [file ACM2-25-e14282-s001.docx]

**Supplementary Table 1** MRI routine examination sequence and parameters of the cervical spine

| **Sequence** | **TR (ms)** | **TE (ms)** | **Slice thickness (mm)** | **Slice gap (mm)** | **FOV (cm)** | **Matrix** |
| --- | --- | --- | --- | --- | --- | --- |
| T_1_WI, Sag | 400–850 | 6–16 | 3–4 | 3.3–4.4 | 8–15 | ≥ 250×190 |
| T_2_WI, Sag | 2000–3500 | 60–140 | 3–4 | 3.3–4.4 | 10–16 | ≥ 250×180 |
| T_2_WI, Axi | 2000–5000 | 80–140 | 3–4 | 3.3–5.5 | 7–10 | ≥ 212×168 |
| FS-T2WI, Axi | 220 | 6.5 | 3 | 3.3 | 7–8 | ≥ 252×249 |

TR, repetition time; TE, echo time; FOV, field of view; FS, fat suppression
